# The role of stents in the treatment of congenital heart disease: Current status and future perspectives

**DOI:** 10.4103/0974-2069.52802

**Published:** 2009

**Authors:** Bjoern Peters, Peter Ewert, Felix Berger

**Affiliations:** Department of Congenital Heart Disease/Pediatric Cardiology, Deutsches Herzzentrum Berlin, Germany

**Keywords:** Stenting, aortic coarctation, pulmonary artery stenosis, patent ductus arteriosus, newer stent designs

## Abstract

Intravascular or intracardiac stenoses occur in many forms of congenital heart disease (CHD). Therefore, the implantation of stents has become an accepted interventional procedure for stenotic lesions in pediatric cardiology. Furthermore, stents are know to be used to exclude vessel aneurysm or to ensure patency of existing or newly created intracardiac communications. With the further refinement of the first generation of devices, a variety of “modern” stents with different design characteristics have evolved. Despite the tremendous technical improvement over the last 20 years, the “ideal stent” has not yet been developed. Therefore, the pediatric interventionalist has to decide which stent is suitable for each lesion. On this basis, currently available stents are discussed in regard to their advantages and disadvantages for common application in CHD. New concepts and designs developed to overcome some of the existing problems, like the failure of adaptation to somatic growth, are presented. Thus, in the future, biodegradable or growth stents might replace the currently used generation of stents. This might truly lead to widening indications for the use of stents in the treatment of CHD.

## INTRODUCTION

Since the first description of an interventional procedure in the field of congenital heart disease (CHD) by Rashkind in 1966,[1] the non-operative treatment of CHD has made enormous progress. While describing percutaneous transluminal angioplasty in 1964, Dotter and Judkins anticipated the need of an endovascular splint to keep the vessel lumen patent,[2] which later resulted in the first implantation of a spiral coil–spring prothesis in experimentally created vascular stenotic lesions.[3] Elastic recoil of vessel wall as well as intimal dissection may lead to acute closure. In these cases, increasing the balloon diameter may worsen the dissection or rupture the vessel. Stents provide more effective and predictable relief of obstruction by exerting a radial force that prevents elastic recoil. They also compress dissection flaps against the vessel wall and prevent acute closure. Stenting may also be helpful in the treatment of extensive vessel dissection after balloon dilatation thus avoiding or ameliorating the risk of aneurysm formation. Long-segment stenoses or hypoplastic vessel segments are not ideal for ballooning. In these cases, implantation of stents may be helpful in achieving long-lasting enlargement to the desired diameter.

Although intravascular stents still do not have the Food and Drug Authority (FDA) approval for use in congenital lesions and pediatric patients, since 1996, their use for congenital vascular lesions has been accepted as the standard of care by all centers and professional medical societies associated with the treatment of pediatric and CHD. This review will address some key questions in the common practice of the use of stents in the CHD population. Advantages and disadvantages of the different types of stents and stent designs currently used in CHD are presented. Furthermore, recent developments in stent technology intended to overcome some of the present problems in stent therapy that might expand the spectrum of indications for stenting are addressed. As this paper focuses on stent therapy within the heart and the central great vessels, the use of stents in peripheral arteries and veins is not discussed.

## COMMON INDICATIONS FOR STENTING

Common indications for stent deployment in CHD include treatment of obstructive lesions of the (1) branch and peripheral pulmonary arteries, (2) systemic and pulmonary veins, (3) aorta and branches, (4) right ventricular outflow tract (RVOT) conduits, (5) maintenance of patency of the arterial duct in duct-dependent circulation, (6) maintaining patency of stenosed aortopulmonary collateral vessels or surgically created but obstructed shunts and (7) maintaining patency of intracardiac communications.

## MOST COMMONLY USED STENTS IN CONGENITAL HEART DISEASE

Stents are classified on the basis of the material of which they are made, the target region, their configuration and their size. Additional features used in classifying them include coverage, special surface treatment and coatings and drug-eluting properties. But, the most common classification is based on their delivery mechanism: Balloon-expandable versus self-expandable stents.

### Balloon-expandable stents

Balloon-expandable stents (BES) are mounted on balloons, positioned across the site of obstruction and are implanted by inflating the balloon. The size of the inflated balloon determines the expanded diameter of the stent. BES were introduced for the treatment of congenital heart lesions in 1987,[4,5] with the development of the Palmaz stent. Since then, they have been the most commonly used stents in pediatric cardiology. The Palmaz stent consists of a 316 L stainless steel tube with laser-cut slots that form seven cells per row. The tremendous success of BES is due to its unique features that combine high radial force with accurate stent placement and oversizing properties (up to 10 mm). With its increasing use and the widening spectrum, some unfavorable characteristics have come into focus. They include marked foreshortening, rigidity, flaring of the sharp edges, vessel injury or balloon rupture and the unsatisfactory bending performance. These limitations have prompted development of new stent designs.

### The “ideal” stent in congenital heart disease

Desirable features for an ideal stent design in pediatric cardiology are: (1) low stent profile combined with (2) high trackability and (3) flexibility to negotiate steep curves, (4) good radio-opacity and visibility for precise placement, (5) compatibility with magnetic resonance imaging (MRI) with no artifacts, (6) predictable expansion with minimal foreshortening, (7) sufficient radial strength, (8) low rigidity with no material fatigue over time, (9) full biocompatibility with resistance to thrombus formation and corrosion, (10) prevention of plaque protrusion, (11) avoidance of neointimal proliferation, (12) round and soft edges for avoidance of intimal damage, (13) possibility of redilation with patients' growth, (14) wide struts to maintain blood flow to jailed vessel branches and (15) retrievability and possibility of repositioning if needed. Obviously, there is no stent yet available that combines all of these requirements so that selecting the right stent for the appropriate condition is one of the most difficult challenges encountered by pediatric interventional cardiologist.

### Design characteristics of balloon-expandable stents

Technically speaking, stent performance is related to the material characteristics, form, fabrication mode and geometry.

### Materials of balloon-expandable stents

The most widely used material for BES is still stainless steel, typically 316 L. It is particularly corrosion resistant and in its fully annealed condition, easily deformable. Alternative materials for BES used in CHD are platinum alloys (Cheatham Platinum [CP] Stent; NuMED Inc., Hopkinton, New York, USA), tantalum (Strecker Stent; Boston Scientific, Natick, MA, USA) and cobalt alloys (the newly developed Andrastent XL and XXL; Andramed, Reutlingen, Germany). Cobalt chromium alloys are very interesting for use in pediatric cardiology as they allow lower crimping profiles with high radial strength. Recently, biodegradable stents have been introduced that are composed of polymeric substances, magnesium or biocorrodible iron. This new class of stents is discussed in the section “New developments in stent design and future perspectives” at the end of this article.

### Slotted tube design

The vast majority of coronary and peripheral vascular stents are produced by laser cutting from tubing, the so-called slotted tube design. Intricate patterns can be produced using tube sizes from 0.5 mm diameter. BES are cut in crimped or near-crimped conditions, allowing a low profile. In slotted tube stents, those with the more conservative closed cell design need to be distinguished from those with the newer open cell design.

### Closed cell design

**Figure 1:**
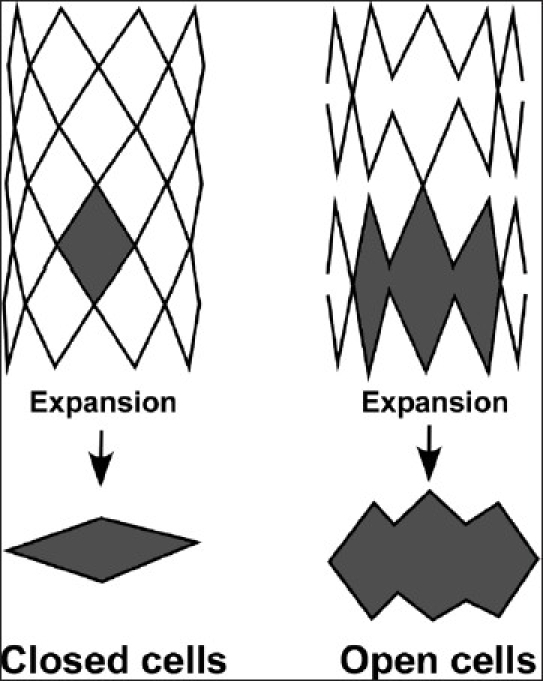
With the closed cell design, cell geometry connects consistently throughout forming complete and bridging cells. With expansion, the individual cells do not merge to form larger open areas. All connections should connect at least three elements. With the open cell design, cell geometry does not connect consistently throughout, forming incomplete and non-bridged cells. With expansion, the individual cells merge to form larger open areas.

The traditional closed cell design describes a sequential ring construction wherein bridging elements connect all internal infliction points of the structural members [Figure 1]. This is only possible with regular “peak-to-peak” connections, meaning that the bridging elements join adjacent structural members at the outer radii. Early slotted-tube type designs, such as the Palmaz stent, were strong but inflexible. Later designs such as the NIR™ stent (Boston Scientific, Natick, MA, USA), the Corinthian™ series (Johnson and Johnson Interventional Systems, Warren, NJ, USA) or the Genesis™ stent (“sigmoidal hinge connection”; Johnson and Johnson) improved upon this concept by adding a flex-connector (U-, V- S- or N-shaped elements) that plastically deforms during bending, allowing adjacent structural members to separate or nest together.

Thus, older Palmaz stents such as the Palmaz 8 series (P 108, P188, P308) and others have been replaced by the Genesis series. The Genesis stents represent the latest evolutionary step of the Palmaz “biliary” stent series. They are similar to the Corinthian stent (a further development of the original Palmaz stent), except for the new sigmoidal hinge. This element adds greater flexibility without compromising radial strength. They are available as unmounted and pre-mounted, the latter for only the smaller diameters – paradoxically named Genesis Medium. The Genesis Medium stents can easily be dilated to 5–8 mm, but overdilation up to 12 mm is possible. Genesis Large (PG295P, PG395P) are labeled as 5–10 mm, but may be dilatable to 12–15 mm. The Genesis XD (XD, extended diameter) can be dilated optimally to 10–12 mm but can easily be overexpanded to 18 mm. Some other commonly used stents with the closed cell design are listed in Table 1.

**Table 1:** Commonly used balloon expandable stents for congenital heart disease

Stent	Open Cell	Closed Cell	Range of diameter	Range of length
Medium stents				
Palmaz 4 series		●	2-4 (-11) mm	10-15 mm
Genesis medium*		●	4-8 (-12) mm	12-24 mm
Genesis large		●	5-10 (-12) mm	29-79 mm
NIR stent		●	4-8 (-10) mm	14-17 mm
Jostent peripheral (large)*		●	6-12 (-16) mm	12-58 mm
Bridge X3	●		5-7 (-14) mm	10-28 mm
Guidant Omnilink*	●		5-10 (-12) mm	12-18 mm
Guidant Herculink	●		5-10 (-12) mm	12-18 mm
Jostent Wavemax	●		4-12 (-14) mm	12-58 mm

Large stents				
Palmaz 8 series		●	4-8 (-20) mm	10-30 mm
Genesis XD*		●	10-12 (-18) mm	19-59 mm
Saxx		●	4-12 (-18) mm	13-80 mm
CP Stent 6 zig		●	6-15 (-18) mm	16-45 mm
Double Strut LD	●		5-8 (-18) mm	16-36 mm
Mega LD*	●		5-8 (-18) mm	16-36 mm

Extra large stents				
Palmaz XL (10 series)		●	6-25 (-28 mm)	30-50 mm
CP stent 8 zig*		●	6-25 (-28) mm	22-45 mm
Maxi LD*	●		5-8 (-26) mm	16-36 mm
Andrastent XL&XXL	●	●	14-32 mm	13-57 mm

The “true” expandable maximal diameter is given in parentheses. The stents are grouped in medium, large and extra large stents.

* stents currently used in our catheterization laboratory

The primary advantage of the closed cell design is optimal scaffolding and a uniform surface, regardless of the degree of bending. However, these advantages result in a structure that is typically less flexible than with a similar open cell design.

### Open cell design

In open cell design, geometry does not connect consistently throughout the stent, forming incomplete and non-bridged cells. With expansion, the individual cells merge to form larger open areas; thus, there is no consistent shape of cells [Figure 1]. Examples for the open cell design are the Guidant Herculink™ and Omnilink™ (Guidant, Santa Clara, CA, USA), Jomed Wavemax™ (Jomed AB, Helsingborg, Sweden), the Medtronic AVE Bridge™ Stent (Medtronic/AVE, Santa Rosa, CA, USA) as well as the IntraStent™ DoubleStrut™, Mega™ LD and Maxi™LD series (EV3 Inc., Plymouth, MN, USA). Stents with open cell design have some characteristics that seem beneficial for their use in CHD: they foreshorten less, are conformable, are less likely to cause balloon rupture and can allow access to jailed branches. These characteristics have been documented for the IntraStent™ DoubleStrut™ (EV3 Inc.).[6,7] This is laser cut out of a 316 L stainless steel tube. The parallel struts and the unique cell geometry allow flexibility around tight curves and minimize stent shortening (especially when serial dilation is performed). While it has similar radial strength to other stents up to diameters of 12 mm when dilated beyond 15 mm, it has less strength.[8] Therefore, in some lesions in which high radial strength is required, these stents may recoil.[6] Thus, if higher radial strength beyond 15 mm diameter is an issue, Intrastent™ Mega™ LD and Maxi™LD stents (EV3) have almost replaced the DoubleStrut stent. In these stents, the configuration of the cells is similar to that of the DoubleStrut stents, but the single struts are cut from a tubing of an initially thicker material, which makes each strut thicker and stronger than the double struts. They have very rounded edges to minimize trauma and their open cell design allows for access to the side branches as the individual cells can be dilated up to 12 mm in diameter. On the other hand, a higher incidence for protrusion of intimal tissue or plaque in between struts has been reported with the open cell design, which might impact long-term stent performance.[7] The Mega™ LD can be dilated up to 18 mm and the Maxi™LD up to 25mm with less than 25% foreshortening when expanded sequentially. Foreshortening can be much higher if these stents are expanded with a single large balloon directly to 15 or 18 mm. The reason for this is that the ends of the stent are compressed toward each other due to the typical “dumbbeling” of the balloon at the end of inflation while the center of the stent is expanding to its full diameter. This results in significant shrinkage of the overall length of the stent. Thus, if final length is critical, the delivery requires sequential balloon dilatation with increasing diameters or, even better, the use of a Balloon-in-Balloon (BIB™ catheter; NuMed Inc., Hopkinton, NY, USA). The recently introduced innovative Andrastent XL and XXL (Andramed, Reutlingen, Germany) are characterized by the so-called hybrid cell design, a combination of closed and open cells, intended to improve flexibility for better delivery performance compared with that of conventional large stents. According to the manufacturer, these stents show significant foreshortening of 48–47% at maximum diameter.

### Welded tube design

In contrast to stents with the slotted tube design, the Cheatham platinum (CP) stent has been developed using a welded tube. It is manufactured from a wire, which is bent and welded to a cylindrical meshwork, forming the stent. This leads to more adjustable flexibility and a wide range of size and length, but radial strength is usually less than the ones with slotted tubes.

The most commonly used stent in pediatric cardiology is the CP stent, which is made from 90% platinum and 10% iridium. Each row of zigs is laser welded with the addition of gold soldering (since December 2002) at each welded spot to increase the total strength of the weld. The number of zigs affects the final diameter and the degree of shortening as well as the profile of the stent. While it is available in 6 and 8 rows, it is mostly used in the 8-zig configuration, as this shortens much less than the 6 zig at the equivalent diameters and can be dilated up to 28 mm (instead of 18 mm for 6 zig), with foreshortening of about 22–28%; however, dilatation to larger diameters is possible.[9,10] These stents have excellent visibility on fluoroscopy and maintain excellent radial strength even at larger diameters. For introduction, sheaths 2 French larger than the recommended sheath size for balloon are required (as they have a slightly larger profile than other stents). They are available in different lengths (22, 29, 34, 39 and 45 mm) but can be customized in every desired length in increments of 6 mm by welding further segments together in longitudinal direction. They have rounded edges, are more malleable and are MR compatible. The rounded edges cause less trauma to the vessels and the balloons. This stent was the first to be developed for exclusive use in pediatric cardiology.[9,10] It carries a CE mark and is now in routine use throughout most of the world except in the United States (the country where it was developed) due to restrictive FDA regulations. In the United States, the CP stent used to be available for compassionate or individual humanitarian emergency use, but even this availability has been suspended. Thus, the Palmaz XL stents are still in use but, due to their “old” stent design (as discussed earlier for the Palmaz 4 and 8 series), those stents have some potential disadvantages compared with the CP and the Maxi LD stents.

### Covered stents

CP stents are available in a covered version, with an outer polytetrafluoroethylene (PTFE) membrane. The covering is initially approximately 7 mm in diameter and will stretch over the range of diameters of expansion (usually from 12 to 24 mm diameter) and will always be taut over the stent when expanded. To date, the covered CP stent is the most widely used covered stent in patients with CHD.[9,11–26]

### Different sizes of balloon-expandable stents for congenital heart disease application

A very practice-orientated classification for the use of BES in CHD is based on the maximum expandable size of stents. It is important to note that, due to the lack of official recognition for use in pediatric and CHD patients, nearly all stent manufacturers test and label their stents with the maximum diameters for use in approved smaller diameter biliary system and peripheral vessels, rather than their true potential maximum diameter. Thus, stents can be categorized into four different sizes by their “true” maximal expandable size: Small (3–6 mm), medium (10–12 mm), large (up to 18 mm) and extralarge (up to 25 mm).[8] Small stents are almost exclusively used in coronary arteries and thus are not discussed here. Medium stents are appropriate for implantation in segmental and subsegmental branch pulmonary arteries, femoral and inominate veins, pulmonary veins and systemic-to-pulmonary shunt (Palmaz 4 series, Corinthian, Genesis medium and large, Guidant Omnilink, Guidant Herculink, Jostent Wavemax, Jostent peripheral, Bridge and NIR). Large stents may be adequate for branch pulmonary arteries, lobar branch pulmonary arteries, the caval vein, proximal iliac vein, aorta, Fontan baffles and (valved) homografts and conduits (Palmaz 8 series, Palmaz Genesis XD, Intrastent Double Strut LD, Mega LD stent and the CP 6 zig). The extralarge stents can be used for the aorta, large right ventricular baffles, Fontan conduits or homografts/conduits (Palmas XL, CP 8 zig, Maxi LD, Andrastent XL and XXL).

In Table 1, we list the BES currently used in pediatric cardiology. Their “true” expandable maximal diameter is in parentheses. Stents marked with an asterisk are currently used in our catheterization laboratory and are considered by us as “modern” stents.

### Special balloons in stenting

The large majority of balloons currently used for stent deployment are still adapted from the adult vascular angioplasty and “biliary” applications. These large- diameter single-balloon catheters tend to expand first at their ends and thereby evert the stent ends such that they protrude radially from the stent center. Deploying a stent in this orientation can cause injury to the vessel wall and may be a risk factor for the development of aneurysm or dissection. One of the most important developments in equipment for the delivery of large-diameter stents has been the Balloon-in-Balloon (BIB™; NuMed Inc.) catheter, the first balloon specifically designed for stent delivery in the CHD population. These catheters have an inner balloon and a longer outer balloon that is double the diameter of the inner balloon. The BIB catheters are available in outer balloon sizes of 8–24 mm. The BIB catheters offer the important advantage of opening the stent more uniformly along its length but require a larger arterial sheath for introduction. With a stent hand crimped onto the balloon, it is necessary to upsize the long sheath by 1F, greater than is necessary for the BIB catheter alone. Therefore, with hand-mounted stents, BIB catheters with outer balloon diameters of 8–14 mm require a 9F sheath, 16 mm catheters a 10F sheath, 18–20 mm catheters an 11F sheath and 24 mm balloons a 12F sheath. Thus, while BIB catheters prevent stent flare and offer more precise control over stent placement, single-balloon catheters are still sometimes preferable in smaller patients to reduce risk of injury to the femoral artery at the access site.

The development of ultra high pressure (UHP) balloons like the Conquest (5–12 mm diameter) and the Atlas (12–26 mm diameter; Bard Peripheral Vascular Inc., Tempe, Ariz) may facilitate successful treatment of stent-associated stenoses that are resistant to conventional high-pressure dilation. These balloons, originally developed and approved for treatment of stenotic hemodialysis fistulas,[27] contain a cross-matrix woven layer of ultrahigh molecular weight polyethylene. They have rated burst pressures ranging from 18 to 27 atm, but can be inflated to substantially higher pressures without rupturing.[28] In a recently published paper, these balloons have been successfully used to treat resistant obstruction within or adjacent to previously implanted pulmonary artery (PA) stents, with successful dilation in 91% of the lesions proven to be resistant with other balloons, and no dilation-related complications.[28] It was even possible to enlarge completely expanded and foreshortened stents by breaking them longitudinally. In our own experience, they may also be helpful when significant residual stenosis remains, e.g. after percutaneous pulmonary valve implantation (PPVI) (Melody™; Medtronic Inc., Minneapolis, MN, USA) following pre-stenting with bare-metal stents (Max LD; EV3, Plymouth, MN, USA). As these balloons are relatively stiff and have a long nose, this may limit their applicability in some circumstances: if they are inflated across a curved or angled vessel, straightening of the balloon at high pressure may result in substantial excursion of the nose, potentially posing a risk of distal vascular injury. Given the very high stresses that can be generated, it is recommended to use a balloon/waist diameter ratio of around 1.2–1.3, which is smaller than that typically applied with standard or high-pressure PA angioplasty.

For lesions resistant to conventional high-pressure angioplasty, evidenced by a persistent waist, cutting balloons may be used before stenting. A Cutting Balloon™ (Boston Scientific, San Diego) has three or four longitudinal microtome blades bonded to a non-compliant balloon. Folded between the material of the balloon, the blades are exposed when the balloon is slowly inflated, achieving a profile of 0.127 mm from the surface. Their microblades are intended to incise only the intima and part of the media in a vessel, resulting in a more controlled dissection of the vascular intima. On deflation, the balloons refold very smoothly and the blades recess back to their original configuration in the surface of the balloon. As they are only available in sizes up to 8 mm, this limits their use in larger vessels. Currently, they have been shown to be very effective in pulmonary branch artery stenosis,[29,30] major aortic-to-pulmonary collateral arteries[31] and enlargement of atrial septal defects (ASDs)[32,33] before stenting.

### Stent mounting

Only a minority of BES suitable for CHD are pre-mounted, like the Genesis medium stent (Cordis) that is available on a Slalom™, OptaPro™ and Aviator™ Balloon (Cordis). The pre-mounting represents a unique incorporation of the stent into the wall of the balloon. This fixes the stent very securely on the balloon and allows delivery of the stents to very circuitous locations without the need for a long sheath and with no displacement during delivery – although using a short sheath access may not always be advantageous, as monitoring of placement with contrast medium injection is more difficult. Currently, most stents for CHD come unmounted and have to be hand crimped over the desired balloon, which also has its own advantages. This offers more flexibility, as stocking is easier because multiple stent–balloon combinations are possible. We sometimes use a customized crimping device (Qualimed, Winsen/ Luhe, Germany) to obtain a very low profile and tight stent/balloon assembly. Thus, it has been possible to deliver a Mega LD stent (EV3) on a 6 mm Powerflex P3™ (Cordis) balloon through a 6 Fr-long sheath (personal experience).

### Self-expandable stents

Materials for self-expanding stents should exhibit large elastic strains. The most widely used material is nitinol, a nickel-titanium alloy that can be recovered from elastic deformations of up to 10%. This unusually large elastic range, commonly known as superelasticity, is the result of a thermoelastic martensitic transformation. The limited elastic range of more conventional materials, such as stainless steel (Cook “Z Stent”) or certain cobalt-based alloys (BSC “WallStent”), offer limited design options. While the WallStent offers excellent wall coverage and flexibility, its shortcoming is its length change during deployment of up to 53.8%, making the newer design [Table 2] more superior.

**Table 2:** Currently used self-expandable stents in CHD. In general indications for self-expandable stents in CHD are scarce

Stent	Diameter	Length	Sheath
Dynalink	5-10 mm	28-100 mm	6 Fr
Protégé GPS	6-14 mm	20-80 mm	6 F
S.M.A.R.T	9-14 mm	30-80 mm	6-7 F
Wallstent	8-10 mm	40-100 mm	8 F
Cook Zilver	6-10 mm	20-80 mm	5-7 F

Most commonly used self-expanding stents in pediatric cardiology include the Wallstent (Schneider, Minneapolis, MN, USA), the S.M.A.R.T™ stent (Cordis Endovascular, Miami, FL, USA), Strecker Stent (Boston Scientific, Natick, MA, USA), Dynalink™ (Guidant, now Abbot Vascular), Symphony (Boston Scientific, Watertown, MA, USA) Cook Zilver™ Nitinol stent (Cook, Bloomington, IN, USA) and the PROTÉGÉ™ GPS™ Stent (EV3 Inc., Plymouth, MN, USA), but there are others on the market. The newer systems demonstrate minor foreshortening of around 8–10% of the original length under full expansion.8

Self-expandable stents, constrained within a covering delivery sheath, are delivered to the site of stenosis. Withdrawal of this sheath uncovers the stent, which then reassumes its original shape (the chosen diameter) to dilate the stenotic lesion. As these stents do not require a balloon for expansion, they can be delivered through a lower-profile delivery system. Compared with BES, they are more flexible and thus can be passed through very tortuous vessels and lesions, but have significantly lower radial strength. They are made from nitinol (nickel–titanium alloy) and are therefore fully MRI compatible. Because of the unique memory effect of nitinol, they show a delayed 10–20% additional expansion within the first month following implantation. This might partially compensate for the development of more neointimal hyperplasia[34] than with conventional BES. This unfavorable increased neointimal induction might be due to a cytotoxic effect of corrosion products of nitinol which have been shown to induce smooth muscle cell necrosis during the *in vitro* study.[35]

Traditionally, they have been used for peripheral vascular applications, where external compression can permanently deform a stainless steel stent, but not the nitinol thermoelastic stent. Their main disadvantage is that they cannot be further dilated to accommodate vessel growth. Therefore, use of this type of a stent in growing children is limited[34–37] unless implanted in structures that are fully grown or do not show any growth potential, such as a surgical shunt, a baffle or a conduit.

Self-expandable stents currently used in CHD are listed in Table 2.

## STENT IMPLANTATION FOR SPECIFIC LESIONS

### Stents in the pulmonary arteries

#### Overview

PA obstructions occur in both congenital and acquired disease and they are found as isolated or multiple lesions, discrete stenosis or diffuse hypoplasia. Genetic syndromes such as Williams or Allagille are associated with such obstructions. They may be part of simple or complex cardiac malformations, such as ventricular septal defect, pulmonary valve stenosis, tetralogy of Fallot (TOF), pulmonary atresia and others. The majority of the acquired lesions are remnants after surgical procedures in complex heart defects. Thus, stenoses may develop as a result of extensive scarring at a previous PA repair site or at the anastomosis between the PA and a conduit or along the aortopulmonary shunt. Sometimes stretching of vascular structures may contribute to formation of stenosis, as seen following the LeCompte maneuver for repair of d-TGA. Such examples indicate that the underlying etiology and thus the pathomechanism of the obstruction differ from case to case. Therefore, optimal therapeutic strategies, based on case characteristics, should be adapted. Universally accepted indications for intervention include symptoms, right ventricular systolic pressure of more than 50% of systemic systolic pressure, right ventricular dysfunction and significant differential pulmonary perfusion in unilateral stenosis. Non-invasive imaging, such as MRI or echocardiography,[38] are useful tools for monitoring patients with suspected problems and can be used to determine the optimal timing for interventions in the absence of symptoms.[38]

The disappointing results of surgical management of branch PA stenoses led to the introduction of balloon angioplasty in the early 1980s.[39,40] With standard balloon angioplasty, an immediate success rate – most often defined as an increase in vessel diameter of more than 50% – of 32-72% has been reported.[41,42] Reasons for acute failure of balloon dilation include resistance to vessel tears or disruption, as evidenced by persistent waist in the balloon, compliant lesions expanding with angioplasty but recoiling to the original diameter and narrowing caused by asymmetric compression from the adjacent structures. Stenoses in the lobar PA branches have been shown to be particularly resistant to conventional balloon angioplasty and are more likely to require the use of high-pressure balloons.[43] During long-term follow-up, restenosis occurs in up to one-third of all patients[44,45] as a result of natural tissue recoil and subsequent scarring. Because of unpredictable and unsatisfactory long-term results following surgery and plain balloon angioplasty, PA stenting was introduced in 1991.[41,46] Subsequent follow-up studies demonstrated that stents maintain long-term vessel patency[47–49] and can be further dilated at subsequent catheterizations.[48,50,51] In the meantime, intraoperative placement of stents under direct vision as a hybrid procedure has completed the spectrum of PA stenting.[52–54]

Indications and use of stents for dealing with PA stenosis have increased in recent times. Stents are mainly used to deal with origin stenosis occurring naturally or after previous surgery, kinking or tenting of the branch PA, external compression of the branch PA, elastic recoil, intimal tear after balloon angioplasty and recanalization of totally occluded vessels. In infants with prior implantation of an RV–PA conduit, stenting is used as a bridge to delay conduit replacement. Although stents are commonly placed after previous balloon angioplasty, there are indications where primary stenting should be considered. These include long-segment stenoses, where balloon angioplasty shows unsatisfactory results,[54] subatretic stenoses, severe forms of PA coarctation, and bifurcation stenting.

### Central PA stenting

There are numerous published papers demonstrating excellent mid- and long-term results[55] after stenting of post-operative or congenital branch PA stenosis.[47–50,55] Therefore, PA stenting is now widely accepted as the first-line therapy of central PA stenoses. If there are additional lesions such as a (residual) VSD, which are not suitable for interventional therapy, a hybrid strategy with intraoperative stenting under direct vision or with fluoroscopic or endoscopic guidance may be beneficial.[52–54] This avoids difficult surgery on fragile pulmonary arteries, shortens time of cardiopulmonary bypass and makes the pulmonary arteries amenable to future percutaneous interventions. The kind of stent to be implanted depends on the anatomic situation, the age and size of the patient and the vascular access status. Usually large stents that may be redilated up to at least 18–20 mm to accommodate somatic growth in children are preferred. Our current choices for central branch stenoses are “modern” low-profile stents like the Palmaz Genesis XD™ stent or the Mega LD™ or the CP stent using a long-sheath access. If the transvenous approach fails due to unfavorable anatomy, a direct access to the branch pulmonary system using a left or right anterior small thoracotomy may be considered for successful stent deployment.[56] In very small children (below 10 kg), a palliative approach may be adequate to avoid early reoperation in complex defects.[57,58] An example would be the implantation of a pre-mounted Genesis medium stent (Cordis) for treatment of early PA restenosis after corrective surgery for TOF. Advantages of these pre-mounted stents are their good deliverability without the need of a long sheath and the very low and flexible profile (5–6 F). Of course, these stents have to be removed later by the surgeon, but stenting can postpone surgery to a later point.[57,58] The presence of a stent in the pulmonary arteries does not seem to interfere with suture lines during subsequent surgery.[52,57] Incising across the stent and suturing through their interstices usually do not present a significant problem to the surgeon. Conduits have been connected to previously stented pulmonary arteries in Fontan completion without problems.[58]

The incidence of significant restenosis of single-vessel PA stents has been reported to be 1.5–7% in large pediatric series.[48,50,55] Two factors are involved: The first is intimal hyperplasia of more than 1–2 mm, particularly at the edges of the stents, at gaps between two stents with diameter mismatch and in lumina < 6 mm; the second is stenosis where no further growth is expected, depending on the initial age and size of the patients. It has been documented by various groups that redilation under these circumstances is a safe and efficient procedure even 10 years after initial stent application.[51,59,60] Considering these data, an intended staged serial dilatation is sometimes advisable to avoid vessel overdilatation that is considered to accelerate neointimal proliferation.

Interventional treatment of bifurcating stenoses in the PA involving either the proximal branch or lobar branch vessels can be technically challenging. Standard balloon angioplasty alone is often inadequate and placement of stents is frequently required to relieve obstruction. In such bifurcating pulmonary stenoses, a simultaneous approach (simultaneous stent placement in two or more adjacent pulmonary arteries) is in our view mandatory and has been described previously.[61,62] The advantage of simultaneous placement is to prevent crushing of the proximal portion of a stent placed in the contralateral vessel, which could cause obstruction to that vessel and make access to the vessel for further intervention quite difficult or impossible.[62] Depending on the size of the vessels involved, “modern” large (Mega LD, Palmaz Genesis XD or CP stent) or extralarge stents are currently used. Our first choice would be the CP stent as it has enough flexibility for good vessel adaptation and has round edges at the “kissing point” of both stents. In very small children, a tightly crimped Mega LD stent (using a crimping device) might be an option as we have been able to deliver this through a 6 F long sheath (on a 6 mm Powerflex P3™ balloon, Cordis). In a recently published long-term study, the restenosis rate was higher in bifurcational stenting (31.8%) compared with singe-vessel PA stenting.[62] The reason for this is unclear but factors such as greater distortion of the vessel walls at the bifurcation after stent implantation, increased exposure of metallic surface of two stents at the bifurcation points or increased turbulence of flow at the bifurcation areas leading to significant intimal proliferation may play a role.[62] Repeat balloon angioplasty or additional stent implantation usually solves the problem.

Distal PA stenoses are usually multiple and represent complex lesions that are not accessible to the surgeon.[63] The smaller pre-mounted Genesis Medium stents (Cordis), although not suitable for more central or proximal branch pulmonary arteries, are very suitable for stenoses beyond the first branching of the right or left PA. With bi/multisegment hilar PA stenoses, a simultaneous approach comparable to the bifurcation stenting is often favored.[6] Therefore, if a side branch arising from a previously stented vessel is stenotic or its origin has been compromised, transcatheter dilation or even stent placement may be necessary. In these circumstances, the open cell design (e.g. IntraStent DoubleStrut LD™, Mega LD™) allows stent placement through the sides (cells) of a previously placed stent.[64] For the smaller pulmonary side branch vessel, pre-mounted stents (Plamaz Genesis medium or larger coronary stents) are usually adequate. If the peripheral PA stenosis is resistant to conventional high-pressure balloons (evidenced by persistent waist), the use of cutting balloons (Boston Scientific, IVT, San Diego, CA, USA) has been shown to be successful. Usually, the intimal dissection caused by the microblades is well tolerated.[29] Although the initial gain in the transluminal diameter is usually maintained during follow-up,[30] some patients still require stent placement.

### Stenting of right ventricular outflow conduits

Failure of a bioprosthetic right ventricle-to-pulmonary artery conduit has been well documented and may require repeat surgical conduit replacement. Pathomechanisms involved are external compression (usually by the sternum), calcification, kinking, development of fibrotic intimal peel and aneurysm. In addition, conduit diameters are limited and young patients may outgrow the diameters. Balloon dilatation of stenotic valves has shown poor results, with minimal extension of the life span of the conduit.[65,66] Thus, stent implantation has been shown to avoid or postpone surgical repair[47,67,68] by accepting high-degree pulmonary insufficiency. In contrast, recently introduced PPVI (Melody™; Medtronic, Minneapolis, MN, USA) treats both pulmonary stenosis and regurgitation with excellent immediate and long-term results[69] and has therefore has replaced the RVOT stent. PPVI is now considered the therapy of choice for stenotic RVOT conduits in selected patients.

### Potential complications in PA stenting

Potential complications with PA stent deployment occur in 4–5% and include stent migration (2.4%), stent malposition, intimal flap obstruction, jailing of side branches, stent fracture, dissection, aneurysm, vessel rupture, hemoptysis (1.5%), thrombosis of stent, ipsilateral pulmonary edema (1.5%) and death (1.5%).[55] In a retrospective long-term survey over 12 years, no mortality or morbidity was observed for the last 4 years due to improvements in technology and material.[55]

## STENTING THE AORTA

### Stenting in coarctation of the aorta

Balloon dilation of aortic recoarctation (Re-CoA) was introduced in 1982 as treatment for post-operative restenosis after surgical repair with subsequent hypertension.[70–72] Intimal and medial vessel wall tears, aneurysm formation, residual CoA and Re-CoA are well known complications encountered at follow-up in patients with residual or native CoA treated with balloon dilation.[73–75] The incidence of early and late aneurysms after balloon angioplasty has been reported to be between 5 and 11.5%.[70,74,76–80] Increasing age has been found to be a risk factor for a suboptimal outcome.[81] This has been attributed to the presence of fibrotic changes in the aorta secondary to long-standing obstruction,[74,77] and cystic medial necrosis observed after percutaneous balloon angioplasty of CoA as potential factors contributing to adverse consequences, such as Re-CoA and aneurysms.[82] Since the early 1990s, BES have been used in the treatment of CoA in children.[10,83–87] The apposition of the torn vessel intima to the media and the ability to perform redilatation to accommodate the patient's somatic growth are the key features. Although they were initially used to reduce vessel wall disruption, aneurysm formation after stent implantation or aortic dissection after stent dilatation have been encountered in up to 5% of the patients.[74,88,89] These complications are inevitable consequences of the mechanism of dilation of the CoA site and so cannot be completely abolished. The perceived advantages of stenting compared with pure ballooning are the ability to expand a tubular long-segment coarctation or an hypoplastic isthmus and distal transverse aortic arch, to increase the diameter of the coarcted segment independent of the intimal tear, to decrease the prevalence of restenosis and to prevent aneurysm formation because of support to the weakened aortic wall segment by the stent. With growing experience in current studies, success rates approach 100%.[10,26,76,81,83,85–87,89–91] In the largest multicenter studies yet published with over 500 patients, acute complications have been reported in up to 14% of the patients. The patients above 40 years represent a high-risk group with 31% incidence of complication. Common complications include vessel disruption,[81,86] displacement of the stent[81,83,86,92] and aneurysm formation.[86,92] Stent migration is the most frequently encountered technical problem, with an incidence of up to 5%.[81,91] Balloon rupture with inadequate stent expansion[81,83] may be prevented by avoiding kinking of the balloon/stent assembly by the use of newer stents with softer ends[81,91] and by the use of BIB systems.[81,91] Injury to access vessels with bleeding from the puncture site[81] (2.3%) is a consequence of the large sheath size required. Bleeding can be avoided in adults, using vascular closure devices like the 6 F Perclose ProGlide™ or A-T™ (Abbott Vascular Devices, CA, USA). With these techniques, safe and effective closure of the femoral access site can be performed up to 14 Fr and even higher.[93] Loss of pulse following femoral vessel closure is of course more likely to occur in patients under 6 years of age.[91] It is our practice, when there is loss of pulse after catheterization, to institute thrombolytic therapy with a bolus of recombinant tissue plasminogen activator (rtPA) that can be repeated after 6 hours Retroperitoneal hematoma due to high puncture point is rare.[81,91] Cerebrovascular accidents have been observed in up to 1%.[81,91] Stent migration is the most frequently encountered technical problem occurring in up to 5%.[81,91] Balloon rupture with inadequate stent expansion[81,83] may be prevented by avoiding kinking of the balloon/stent assembly by the use of newer stents with softer ends[81,91] and by the use of BIB systems.[81,91] Because of post-stenotic dilatation, the stent might not always completely adapt with its distal segments; usually, this is not critical, if good anchoring has been achieved in the proximal half to two-thirds of the stent. Some degree of apposition may be achieved with further flaring of the distal (and proximal) parts with larger balloons, but this carries the risk of stent migration. Sometimes with Re-CoA high-pressure balloons are necessary, but this condition is better dealt with by covered stents (this is addressed later). In severe native or even post-operative CoA, we often avoid full dilatation of the stent at the initial procedure in an attempt to minimize vascular damage, uncontrolled intimal tear and aneursym formation.[9,23] Furthermore, a residual waist better secures the stent in its final position, thereby reducing the risk of stent migration. After complete endothelial healing (about 6 months later), full-stent dilatation to the desired size (usually the diameter of the pre-stenotic aortic arch or the descending aorta at diaphragm level) can be performed.[9,23] Especially in native CoA, which might be relatively elastic, we usually avoid pre-dilatation with a balloon so that better anchoring of the primary implanted stent can be achieved. Aggressive pre-stent angioplasty could also increase the likelihood of later aneurysm formation.[81,91] Large stents are needed for stenting CoA. Traditionally, the large Palmaz stents like the P8 and P10 series are the most commonly used stents in large multicenter studies.[81] Our first choice now is the CP stent[9] or the IntraStent Mega and Maxi LD (EV 3) as those stents are more flexible and can be easily dilated up to adult vessel diameters (20–25 mm) [Table 1]. The Genesis XD (Cordis) stent might also be suitable, but can only be dilated up to 18 mm. The innovate Andrastent – the first peripheral cobalt chromium stent – can be dilatated up to 25 mm in the XL and up to 32 mm in the XXL version. Thus, it may be very suitable for CoA treatment as has been described by De Giovanni (GUCH Unit, University Hospital, Birmingham, UK, personal communication). The chromium–cobalt technology allows very low profile designs and the hybrid closed/open cell design may be very helpful when placing the stent in a curved part of the aorta, but more experience is needed.

### Redilatation in CoA

In general, CoA redilatation demonstrates good intermediate follow-up results.[91,94] Aneurysm formation occurs in up to 9% of all patients, but often reintervention is not necessary. Recurrence of stenosis has been reported in around 11% of all patients. In general, there are two conditions leading to restenosis and late failure after primary successful stent implantation. One is “full-growth” stenosis, depending on the initial age and size of the patient. The other is hyperplasia of the endothelial intima of the vessel. Narrowing of the lumen by 1–2 mm is seen in all cases, but true intimal hyperplasia in less than 25% of patients.[86,94] Risk factors promoting neointimal proliferation include younger age, lower weight and recurrent CoA[86,94,95] as well as stent overdilation, minimal stent overlap and sharp angle between the stent and the vessel wall.[60] Newer stent designs that are more flexible and thus show better adaptation to the vascular wall might induce less neointimal layer formation. In the largest reported series of stent procedures for CoA redilatation (28 patients), 93% success rate has been reported.[96] Four main factors could be identified contributing to the failure of CoA stent redilatation: Severe neointimal hyperplasia, a history of surgical CoA repair, the association of Williams syndrome and the rare occurrence of aortic wall dissection.[96] Drug-eluting stents (Koch, Ludwig *et al*. 2007; Riede, Schneider *et al*. 2007; Jhang, Chang *et al*. 2008; Wong, Yoo *et al*. 2008) or systemic administration of anti-proliferative drugs such as everolimus may theoretically overcome some of these problems,[97] but no sufficient data have yet been published. In conclusion, redilatation is unproblematic, safe and effective but limited by the maximal stent diameter.[96]

### Covered stents and self-expandable stent grafts in CoA

In 1999, the first covered stent was used to treat coexistent CoA and aneurysm of the aorta in a young man.[98] Aneurysm formation, which has been described after both surgical[99] and endovascular treatment and dilatation,[100] is clearly associated with increased risk of aortic rupture. Covered stents[9,11,12,17,18,22,25,98] or endoluminal stent grafts[101–103] have been successfully used in the management of aortic aneurysm in an effort to reduce the need for repeat surgery. The main disadvantage of stent grafts (e.g. the Valiant™ endoprosthesis; Medtronic, Minneapolis, MN, USA) is that they require large (22–25 F) vascular sheaths,[12] in contrast to the use of 12–16 F Mullins-type delivery sheaths with the most widely used covered CP stent.

Aneurysm formation may occur even after redilatation of a previously implanted uncovered stent;[96] the stent design and the aggressive protocol of redilatation might play a decisive role. However, the availability of covered stents can help to overcome this complication. Another potentially catastrophic complication of balloon angioplasty and stenting in CoA is aortic disruption[88,104] and the risk is increased in elderly patients.[81] It may even occur after covered stent implantation.[11,105] Aortic disruption may be managed in an emergency by the deployment of a (additional) readily available covered stent.[11,105] Stent fracture is a well described but uncommon complication after CoA stenting, affecting even the CP stent with reinforced welded sites.[9,18] It may be associated with recurrent CoA. In the case of a fractured stent disrupting the aortic wall at the level of the fracture margins, the use of covered stents could potentially seal any endothelial disruption.[11,18] Besides emergency or rescue indications, covered stents for CoA are being preferentially deployed in the treatment of associated aneurysm, tight/subatretic native CoA,[13,17,18,23] associated arterial duct or collateral vessel[17,18,21,24] and in older patients, due to the potential for aortic disruption with advanced age.[11,18,22,26,90] They have been also recommended for patients with associated prominent dilatation of the aorta, suggestive of an aortopathic process like in bicuspid aortic valve.[12] Some operators have gone as far as to exclusively use covered stents in preference to bare stents in all cases of adolescent and adult CoA and Re-CoA, although this is not yet common practice. Theoretically, the ability to fully expand the covered stent while providing a degree of protection in the event of aortic wall disruption allows optimum dilatation of the stent thereby preventing migration and decreasing the risk of vessel recoil and recurrent CoA. To date, the covered CP stent is the most widely used covered stent in pediatric and CHD patients.[9,11–26] It may be used in children as redilatation is possible to accommodate somatic growth.[15,16] Use in children is, however, limited due to the bigger sheath access, with increased risk of vascular injury. Thus, the sheath has to be chosen 3 F larger than required for the introduction of the balloon catheter, which is one reason why covered stents are currently used mostly in the adult size population. Our current benchmark with the CP stent in CoA is weight of around 15 kg.

One obvious limitation of covered stents is their potential to cover side branch vessels during deployment, usually the subclavian artery ostium. Jailing of subclavian artery is usually well tolerated without functional deficit but an intact vertebrobasilar system should be documented to avoid subclavian steal.[10,92] To circumvent complications in the case of insufficient “aortic margin,” uni- or even bilateral surgical reimplantation of the subclavian arteries end-to-side into the carotid arteries should be considered before stenting as this creates a stable “landing zone” for subsequent stent deployment.[12] Recanalization by perforating the covering of the CP stent followed by balloon angioplasty or side branch stenting might be another option to access trapped side branches.[106] Occlusion of side branches distal to the coarctation site may be more serious with spinal artery occlusion leading to paraplegia, as previously described using long stent grafts.[107] However, the spinal artery usually originates below the level of the ninth thoracic vertebra from the aorta below the diaphragm;[108] therefore, spinal artery occlusion is unlikely to occur. But, operators should keep in mind that stent migration with fixation of the dislocated stent by redilation in a smaller distal area of the aorta as a rescue procedure might potentially occlude critical side branches that it crosses.

Although covered stents are primarily used for CoA treatment, they might be applied to other vessels. Thus, the use of a covered 8-zig CP stent for treatment of post-operative RVOT has been reported[109] Baffle leaks associated with baffle obstruction after atrial switch procedure may be successfully abolished.[14] One of the most innovative concepts is the “rebuilding” and “creation” of an internal venous tunnel with long and covered CP stents for completion of Fontan circulation in the catheterization laboratory.[20] Although the use of covered stents in the pediatric population is restricted by the need for larger sheaths, covered CP stents may be successfully applied in growing children. It has been demonstrated that those stents may be redilated up to 25 mm diameter as the ePTFE covering is quite abundant, very elastic and does not induce an inflammatory reaction.[15,17–19]

### Complex aortic arch lesions

With increasing experience in CoA stenting, the concept has been extended to the treatment of complex stenoses of the aortic arch and the descending aorta.[9,17,110] It has been shown that stenting of complex aortic arch lesions through the whole aortic arch can be performed safely and effectively with excellent, immediate and midterm results. Holzer *et al*. described an improvement to more than 90% of the “normal” adjacent aortic arch.[110] Patients with weight below 10 kg or after hybrid stage I palliation may be at increased risk of adverse events such as peripheral vascular complications or hemodynamic instability requiring administration of inotropes and/or chest compression.[110] Because of anatomical restrictions, overstenting of major arch vessels may be necessary, but is usually not problematic, as long-term follow-up demonstrated no compromise of distal perfusion in otherwise normal vasculature.[9] We primarily use the 8 zig CP stent, usually uncovered, as it has sufficient radial strength, flexibility and large cells to allow side branch flow.[9] The availability of the stent in many different lengths allows excellent adaptation to individual aortic anatomy and enables its use even in small children.[9] Alternatively, the Mega and Maxi LD (EV3) offer good options as they have even more radial strength with similar flexibility. The open cell design favors side branch access and, due to its low profile, sheath size 1–2 F less may be needed than for the CP stent. Stenting of trapped vessels through the stent cells is usually possible, if needed.[7,64]

### Stents in the systemic venous system

Several conditions may lead to stenosis of systemic veins, including superior and inferior caval vein obstruction occurring after atrial switch operation or cardiac transplantation, uni- or bidirectional Glenn anastomosis and total cavopulmonary connection, correction of anomalous pulmonary venous connections and the presence of transvenous pacing electrodes or central venous catheters.[111–113] As balloon dilatation may not be effective in these lesions, stent implantation represents an attractive treatment concept that was introduced nearly 20 years ago.[111] Usually, the presence of symptoms in association with a stenotic lesion makes an intervention necessary. Baffle obstruction following atrial switch operation (Mustard and Senning operation) is the most common indication for stent delivery in the venous system. Most studies show favorable results with relief of clinical signs of obstruction and improvement in pressure gradients and angiographic appearance.[14,37,112–126] We successfully recanalized an atretic superior caval vein with two covered 8 zig CP stents in a Mustard patient with transvenous pacing leads. Complete relief of superior caval vein congestion could be achieved. The use of covered (CP) stents should be considered in subatretretic or atretic lesions[117,127] or if additional baffle leaks are present.[14] These stented vessels may accommodate newly placed pacing electrodes, but this carries a higher risk of recurrent stent occlusion.[128] Pulmonary venous baffle obstruction might also be addressed by stent therapy, but is technically more challenging.[119,126] Rarely, patients who have undergone orthothopic heart transplantation may present with stenosis at the site of venous anastomosis that can be successfully treated by stent therapy.[129] Baffle fenestrations in total cavopulmonary connection may be closed using covered stents,[130] especially in the presence of a baffle stenosis.[123] The stenting of central pulmonary vessels after Glenn or Fontan procedure represents a very special situation as passive flow in the pulmonary arteries depends on optimal flow conditions. In those cases, any obstruction within the pulmonary circulation should be definitely abolished.[9] Although the use of self-expanding stents has been reported,[37] BES has become the treatment of choice for these systemic venous obstructions. We prefer 8 zig CP stents and those with modern open cell designs like the Mega or Maxi LD series (EV3) because they are less rigid, have smoother margins compared with older designs and are thus less traumatic to the thin venous vessel.

### Stents for pulmonary vein stenosis

Pulmonary venous stenosis may be congenital or acquired after cardiac surgery. It is reported after repair of anomalous pulmonary venous return in about 10% of the patients.[131] Sutureless marsupialization that avoids any stitches in the cut edges of the pulmonary veins is now considered the best surgical approach,[132] but freedom from reoperation or death at 5 years is still only ≈50%. These stenoses are almost resistant to balloon angioplasty,[132,133] although the use of cutting balloons (Boston Scientific, IVT, San Diego, CA, USA) may be beneficial.[134] Stenting initially seemed very appealing, but restenosis has been universal.[132,135,136] As the transcutaneous approach may be difficult, especially in small children, intraoperative placement of stent may be an alternative mode of delivery.[52,137] Unless stent placement is considered to be a strictly temporary measure, only stents that allow future expansion to adult dimensions (> 12 mm) should be used. Therefore, stents like the pre-mounted Genesis medium stents (Cordis) may be a good choice. Compared with secondary stenosis, the so-called “congenital pulmonary venous stenosis” is an entity of its own, with an even more malignant course. Long-term results of surgery and stent treatment are very disappointing. Thus, stent therapy usually is of a palliative nature[52,132,138,139] and may serve as a bridge to lung transplantation.[140]

### Stents to ensure patency of intracardiac communications

For some complex congenital heart defects, an unrestrictive ASD is essential for adequate blood mixing at the atrial level or relief of right or left atrial hypertension to achieve an adequate cardiac output and/or systemic saturation. Transcatheter creation or enlargement of an ASD is usually achieved employing techniques including transeptal puncture or radiofrequency perforation (in the case of intact septum), balloon or blades septostomy and static balloon dilatation of the interatrial septum (IAS). However, due to the increased thickness of the IAS in infants beyond the neonatal period or in neonates with hypoplastic left heart syndrome, the use of those techniques may be ineffective to achieve and maintain an adequately sized communication. Therefore, atrial septal stent implantation has been successfully applied to promote a durable unrestrictive ASD.[32,33,141–145] The prior use of cutting balloons (Boston Scientific) may be helpful,[32,33] as the microsurgical blades of the cutting balloon allow controlled tearing of the septal wall rather than stretching of the thickened ASD as seen with static balloon dilation.[33,145] We and other authors in most cases use pre-mounted Palmaz-Genesis stents (Cordis; Johnson and Johnson, Miami, FL, USA) ranging from 7 to 8 mm in diameter and from 12 to 26 mm in length. Sometimes shaping the stent by redilating with a bigger-sized balloon to create a “diabolo” configuration secures the stent and avoids stent migration. Close follow-up is necessary as thrombus formation and progressive obstruction may occur.[32] Failing Fontan circulation is another modality where fenestration may be beneficial. Thus, the use of covered and non-covered stents has been described to maintain patency of the newly created communication.[146–149] Covered stents may have the potential to reduce the acute risk of bleeding.[148]

### Stents in the arterial duct

In duct-dependent CHD, ensuring patency of the arterial duct may be a life-saving procedure. Stenting the arterial duct in defects such as pulmonary atresia, right ventricular hypoplasia, critical pulmonary stenosis, TOF and other complex lesions with reduced pulmonary blood flow may avoid (emergency) surgical shunt. The concept of stenting the ductus arteriosus to increase pulmonary blood flow in cyanotic neonates began with the arrival of stents designed for coronary arteries in the early 1990s.[150] Unfortunately, this strategy lost favor due to delivery systems being relatively stiff and articulated stent designs that resulted in early restenosis.[151] With the availability of new stent designs and catheter technology (low profile, flexible, pre- mounted stents with good scaffolding), better patient selection and preparation, optimal interventional access and covering of the complete length of the duct, results have significantly improved.[152–156] Transcatheter procedures have an advantage with respect to subsequent surgery since there is no scar tissue in the chest from previous thoracotomy or sternotomy. Usually the stent itself presents little surgical challenge. In our recent practice, we have opted for ductal stenting in the presence of special circumstances such as severe hypoxemia unresponsive to conservative treatment, unfavorable anatomy with ductal coarctation, children of Jehovah's Witnesses or others. In addition, implantation of stents represents an attractive alternative for reoperation in neonates and infants with acute complications after surgical creation of a systemic-to-pulmonary arterial shunt.[157] In most cases, balloon-expandable bare metal stents are utilized. For duct-dependent pulmonary circulation lesions, the stent should be expanded to 3.5–5 mm depending on the size of the patient. Any coronary stent (e.g. Liberté stent; Boston Scientific, Natick, MA, USA) or other pre-mounted low-profile stent can be used. The Palmaz Genesis medium stent is currently our first choice. Overlapping may be necessary to cover a long and tortuous duct. In the case of duct-dependent systemic circulation – usually in the hybrid approach for hypoplastic left heart syndrome – a stent size of 6–10 mm might be necessary. Therefore, self-expanding stents (e.g. PROTÉGÉ™ GPS™ Stent, EV3) are generally preferred.[158,159]

### RVOT stenting as a pallitative treatment

In severe forms of TOF with PA hypoplasia, critical RVOT obstruction and the presence of MAPCAs major aortopulmonary collateral arteries (MAPCA), primary repair may not be possible. For such a two-staged repair, early surgical options include a central aortopulmonary anastomosis, a modified Blalock–Taussig anastomosis, an RV outflow patch or ductal stenting as discussed in the previous section. In selected cases, RVOT stent implantation may be another alternative to surgical palliative treatment in order to achieve sufficient oxygen delivery and to induce PA development through pulsatile flow.[160,161] RVOT stenting may also be considered after high frequency (HF) perforation in pulmonary atresia.[162] Because of the small size of these children, pre-mounted coronary or biliary stents like the Palmaz Genesis medium on Slalom balloon are preferred.[161] Although many of these stents cannot be dilated to adult size (coronary stents usually up to 4-6 mm, depending on the type, the Palmaz Genesis up to 12 mm), their efficacy in small infants (even premature[163]) and children in whom further surgery will ultimately be required makes them desirable for this group of patients.[57,161] Usually stents that are located in the RVOT are easily removed at the time of conduit replacement.[57] Those in the proximal PA can also be removed or cut open allowing for PA augmentation.[57] We successfully transferred the palliative concept of RVOT stenting in very small children to an adult patient (27 years) with pulmonary atresia, VSD and MAPCAs. After HF perforation of pulmonary atresia, several CP stents were implanted in the newly opened RVOT and a significant growth of the central pulmonary arteries could be achieved. The high forces from the hypertrophied muscular RVOT led to multiple stent fractures – a common feature with RVOT stent[164,165] – resulting in loss of stent integrity, insufficient scaffolding of RVOT and subsequent restenosis so that serial stent-in-stent implantation was necessary. In total, we implanted four CP stents (two covered, two uncovered, maximum length 55 mm) over a period of 28 months. Thus, a communication of 20 mm diameter between the right ventricle and the PA was established, allowing the interventional closure of two MAPCAs with competitive flow. The development of the central pulmonary arteries with this concept was a tremendous success so that the patient is now awaiting corrective surgery. Although not observed in our patient, stent fragment embolization within 2 months of implantation has been described and vigilant follow-up is recommended for RVOT stents.[164]

### Stenting aortopulmonary collateral arteries

Stenosis of aortopulmonary collateral arteries is common and frequently progressive in patients with unrepaired pulmonary atresia and ventricular septal defect, leading to severe hypoxemia and limitation of exercise capacity in up to 58–68% of the patients.[166] Surgical creation of a new aortopulmonary shunt may provide short-term palliation but may be associated with significant morbidity and mortality. Balloon dilation alone is usually not helpful as it produces minimal change in vessel caliber and no improvement of flow.[167] Therefore, stenting of stenosed aortopulmonary collaterals has become an attractive alternative resulting in significant clinical improvement.[167–169] As these stenoses tend to be extremely hard and thus resistant to high-pressure balloons, the use of a cutting-balloon catheter before stenting might be helpful.[31] Pre-mounted stents with high radial force (e.g. Genesis medium stents, Cordis) are necessary. We have observed radial stent fractures requiring repeat stent-in-stent implantation. The rate of complications is low but includes vessel rupture with pulmonary hemorrhage, dissection, aneurysm formation, vasospasm and occlusion of the arteries.[31,167–169]

### New developments in stent design and future perspectives

There are some recent developments in technology intended to overcome some of the disadvantages of currently used stents.

## BIODEGRADABLE STENTS

### Overview

As scaffolding is necessary to overcome late vessel remodeling only for a limited period – approximately the first 6 months following stent implantation – the concept of biodegradable stents has emerged. In addition to other advantages, this concept might solve one of the main problems in pediatric stenting – the adaption to growth. These systems should deliver a temporary longitudinal and radial straightening effect, offer better physiologic repair, allow reconstitution of local vascular compliance and should not restrict surgical or interventional revascularization thus allowing the possibility of growth and late positive remodeling. The ideal characteristics of a biodegradable stent have been defined as (1) sufficient radial strength to prevent vascular recoil, (2) minimal thrombotic and inflammatory response, (3) avoidance of intimal proliferation, (4) reabsorption of stent components within months, (5) no release of toxic products or embolic material during breakdown and (6) easy processing and sterilization. Based on these considerations, two different concepts have evolved: Absorbable polymer stents and absorbable metal stents.

### Polymeric stents

There are several polymeric degradable stents in development but just a few merit mention at this stage on the basis of clinical data. The BVS stent (Abbott Laboratories, Abbott Park, IL, USA) is made from poly-L-lactic acid (PLLA) and results from clinical trials were recently published.[170–172] While the study demonstrated feasibility, it was a very small patient population (30 patients) and the degradation rate was slow (more than 1 year), which some consider to be one of the drawbacks of polymeric biodegradable stents. One of the earliest devices in this field was the Igaki-Tamai coronary stent (Kyoto Medical Planning Co., Kyoto, Japan), which is also manufactured from PLLA with a zigzag helical coil design.[173] A small trial (15 patients) with 6 months follow-up showed no pronounced intimal hyperplasia, unlike in stainless steel stents. Interestingly, there was evidence of vascular remodeling at the stented site, with an increase of the stent cross-sectional area associated with a decrease in the lumen cross-sectional area, although after the third month no further stent expansion was observed.[173] Although these promising findings demonstrated feasibility and safety, with acceptable efficacy in human coronary arteries, polymeric biodegradable stents have not yet been used in the pediatric population.

### Magnesium alloy stents

The relative ease with which magnesium corrodes and its role as an essential element in the biological system makes it an excellent candidate for the biocorrosion concept. First data was published in 2003.[174] As an alternative to biocorrodible irons, magnesium alloys may offer several advantages. Their use has been tested in several trials in coronary arteries: Progress Trial[175] and BEST-BKT Trial.[176] The first report of implantation of absorbable metal stents in human use was in 2005, in a case of compassionate use to treat left PA stenosis as a hybrid approach, an absorbable magnesium stent (AMS, Biotronik™, Germany) was implanted by Zartner and coworkers in a premature newborn. It showed only mild intimal proliferation with no stent-related inflammatory reaction.[177,178] Later, Schranz and coworkers demonstrated the use of the same stent for acute treatment of a newborn with severely impaired heart function due to a long-segment Re-CoA after complex surgical repair.[179] In this patient, early restenosis was due to accelerated degradation. Similarly, significant restenosis was observed 4 months after implanting a biodegradable magnesium stent within a stenotic aortopulmonary collateral in a 2-month-old girl with pulmonary atresia.[180] Another major disadvantage of AMS is the lack of radioopacity, which means that the stent is not visible during positioning, expansion and after balloon retrieval, which might cause difficulties in the detection of embolized stent part and in precise placement of overlapping stent segments.[177–179] Despite these drawbacks, AMS is an attractive and promising future concept.

### Iron stents

Limitations posed by biodegradable polymer and magnesium alloy stents, such as chronic inflammation and premature recoil, might be overcome by biocorrodible iron stents. The NOR-I Stent is a pure iron stent that has been implanted in an animal study, demonstrating low thrombogenicity, mild inflammatory response and no toxicity, no pronounced intimal hyperplasia and excellent patency, but a very slow degradation rate of several years.[181,182] In addition, *in vitro* experiments showed that ions released from iron stents might reduce the proliferation rate of vascular smooth muscle cells by influencing growth-related gene expression and could therefore play a beneficial role in antagonizing restenosis.[183]

### Limitations of biodegradable stents

All of the degradable technologies reviewed here have many specific challenges ahead of them, but, to date, common to all is the lack of clinical evidence demonstrating a clear advantage of this approach. Polymer systems face specific challenges in achieving adequate strength and resistance to recoil as well as a need to reduce degradation times. Magnesium systems appear to offer the most promise, with degradation times being closer to what may be required. Iron stents face big difficulties in terms of establishing that the degradation products would be acceptable.

### Growth stent

Another interesting approach to overcome the “growth” problem is the development of the so-called Growth Stent.[184] It is made of two separate longitudinal halves of laser-cut and electropolished 0.16 mm stainless steel. The halves are connected with bioabsorbable sutures (Polydioxanon 7-0, PDS II, Ethicon, Norderstedt, Germany) to create a circular stent. The reabsorbable sutures will be completely absorbed after about 6 months. The stents are pre-mounted on dilatation balloons of 4–8 mm diameter (Tyshak II, Mini Ghost, or Tyshak Mini, NuMed Canada, Cornwall, Ontario) and they fit through a 5 F introducer sheath.[184,185] After demonstrating feasibility of this “open-ring” stent concept within an initial animal study,[184] we successfully evaluated its clinical use in eight patients with aortic (Re-)CoA and four patients with stenosis of the aortic anastomosis after a Norwood I procedure. There was only one case of CoA in which early redilatation 3 months after implantation due to pronounced intimal hyperplasia was necessary. Six patients received a large stent after 19–34 months. In light of this promising data, a merely interventional concept for the treatment of native CoA in infants without the need of any surgery might evolve. Thus, the Growth Stent can be used as an effective bridge for infants to gain time until they have reached a body weight of 12–14 kg and can receive a larger conventional stent that will be able to be redilated in the future to adult sizes, i.e. larger than 20 mm in diameter (we recommend uncovered and covered CP stent or Mega LD™). Of course, long-term follow-up with more patients is needed and the results have to be compared with those of surgical therapy, still considered the gold standard in neonatal CoA. In the meantime, some of the observed problems, such as the absence of adaptive growth of the stented area, have to be addressed.

### Surface processing and modification

Electropolishing is the state-of-the-art finishing process for nitinol implants, such as stents. Several studies have shown that electropolished nitinol surfaces exhibit better corrosion resistance, biocompatibility and overall surface quality than surfaces finished by other methods.[186,187] Thus, the Growth Stent (Qualimed, Winsen/ Luhe, Germany) is finished through electropolishing. Such surface modifications might also lead to better biocompatibility of stents in CHD use, but currently no published studies exist. Another form of surface processing is the coating with drugs. The potential benefit of drug-eluting stents (DES) is attenuation of neointimal proliferation, which has been extensively described in adults with coronary arterial disease.[188] For use in CHD, implantation of DES has been reported in the arterial duct feeding an isolated left PA,[189] a conduit in the RVOT of an infant,[189,190] the vertical vein of a supracardiac totally anomalous pulmonary venous connection,[191] an obstructed infracardiac totally anomalous pulmonary venous connection[192] and a pulmonary vein stenosis after orthotopic heart transplantation.[129] As most of the experience with DES comes from coronary stents in adults, the efficacy and safety of these devices in CHD and for pediatric use remain unknown. However, if the mechanism of restenosis is the same in coronary arteries and other vessels, these devices may hold future options for children with CHD.

## CONCLUSION

Over the last 20 years, the usefulness of stents in patients with CHD has become well established. Stent implantation is now considered to be a safe and effective method in relieving a wide variety of pre- and post-operative vascular stenoses not amenable to pure balloon dilatation. Thus, stent therapy is now considered to be the standard and first-line treatment for CoA in adults or post-operative PA branch stenoses. The majority of stent applications are intended to provide a long-lasting cure or reasonable palliation to avoid high-risk repeat operation. Thus, stents may now be used to ensure patency of naturally occurring (e.g. ASD or arterial duct) or artificially created intercirculatory communications. Because of a tremendous improvement in stent technology, the first-generation models like the original Palmaz stent have now been replaced by “modern” designs. But still the device that meets the requirements of an ideal stent for use in CHD patients remains to be developed. Thus, concepts for stent application in very small patients that allow adaptation for somatic growth until adulthood vessel size is reached are clearly needed. Another issue to be addressed is improvement of biocompatibility to avoid intimal hyperplasia and restenosis. Until these problems are solved, the crucial question in this interventional field still remains: Which stent(s) should we use for which lesion? To clarify these questions, anecdotal data are insufficient, underlying the clear need for large prospective trials to search for objective performance criteria. As the population with CHD is limited, independent objective multinational registries are needed. With these improvements, indications for stent treatment in CHD are likely to widen in the future.
